# The impact of analytic method on interpretation of outcomes in longitudinal clinical trials

**DOI:** 10.1111/j.1742-1241.2008.01808.x

**Published:** 2008-08

**Authors:** A Prakash, R C Risser, C H Mallinckrodt

**Affiliations:** Lilly Research Laboratories, Eli Lilly and Company,Indianapolis, IN, USA

## Abstract

**Aims:**

Various analytical strategies for addressing missing data in clinical trials are utilised in reporting study results. The most commonly used analytical methods include the last observation carried forward (LOCF), observed case (OC) and the mixed model for repeated measures (MMRM). Each method requires certain assumptions regarding the characteristics of the missing data. If the assumptions for any particular method are not valid, results from that method can be biased. Results based on these different analytical methods can, therefore, be inconsistent, thereby making interpretation of clinical study results confusing. In this investigation, we compare results from MMRM, LOCF and OC in order to illustrate the potential biases and problems in interpretation.

**Methods:**

Data from an 8-month, double-blind, randomised, placebo-controlled (placebo; *n*= 137), outpatient depression clinical trial comparing a serotonin-noradrenalin reuptake inhibitor (SNRI; *n*= 273) with a selective serotonin reuptake inhibitor (SSRI; *n*= 274) were used. The study visit schedule included efficacy and safety assessments weekly to week 4, bi-weekly to week 8, and then monthly. Visitwise mean changes for the 17-item Hamilton Depression Rating Scale (HAMD_17_) Maier subscale (primary efficacy outcome), blood pressure, and body weight were analysed using LOCF, MMRM and OC.

**Results:**

Last observation carried forward consistently underestimated within-group mean changes in efficacy (benefit) and safety (risk) for both drugs compared with MMRM, whereas OC tended to overestimate within-group changes.

**Conclusions:**

Inferences are based on between-group comparisons. Therefore, whether or not underestimating (overestimating) within-group changes was conservative or anticonservative depended on the relative magnitude of the bias in each treatment and on whether within-group changes represented improvement or worsening. Preference should be given in analytic plans to methods whose assumptions are more likely to be valid rather than relying on a method based on the hope that its results, if biased, will be conservative.

**What's known**

Missing data and the bias it can cause are almost ever-present concerns in clinical trials. The last observation carried forward (LOCF) and observed case (OC) approaches have been common methods of handling missing data in clinical trials and are often specified in conjunction with analysis of variance (anova) to assess longitudinal outcomes, despite the fact that their use entails restrictive assumptions that are unlikely to hold true. Considerable advances in statistical methodology and in our ability to implement those methods have been made in recent years. More principled approaches that require less restrictive assumptions than LOCF and OC have gained widespread acceptance because they are more robust to the biases from missing data than LOCF and OC, and therefore provide better control of false-positive and false-negative errors. One of the newer methods, increasingly referred to in the literature as MMRM (mixed model for repeated measures), has been studied extensively in the context of clinical trials.

**What's new**

Although the performance of MMRM compared with LOCF is well characterised in the literature, the emphasis has been on acute efficacy outcomes. With the increased popularity of MMRM, it is also important to characterise results from MMRM, LOCF and OC in safety outcomes and in long-term studies. This investigation compared results from efficacy and safety outcomes in a long-term clinical trial in major depressive disorder, thereby illustrating how the benefits of more robust analyses such as MMRM can improve our understanding of the risks and benefits of drugs.

## Introduction

Treatment effects are often evaluated by comparing change over time in outcome measures vs. placebo or an active control. However, valid analyses of longitudinal data can be problematic, particularly when some data are missing for reasons related to the variable being analysed (1,2). Since the problem of missing data is almost ever-present in clinical trials, numerous methods for analysing longitudinal data and handling missingness have been proposed, examined and implemented (1–31).

Analyses of mean changes from baseline in clinical trials have traditionally relied on simple methods such as analysis of covariance (ancova) with missing data imputed by carrying the last observation forward (last observation carried forward, LOCF) or by including only completers – those patients who had an observation at the end-point visit (observed cases, OC). However, these approaches entail the restrictive assumption that there is no relationship between either the observed or unobserved outcomes for the variable being analysed and the probability of dropout. This assumption is referred to in the statistical literature as missing completely at random (MCAR). In an efficacy analysis, this assumption essentially means that patients do not drop out for lack of efficacy. The LOCF approach further assumes that subjects’ responses would have been constant from the last observed value to the end-point of the trial.

These assumptions may not hold true in clinical trials (6,12,13,19), and violations can confound treatment with time (2), which in turn can bias estimates of treatment effects and their standard errors (SE) (2,6,9–13,19,21,22,25–27). It is often assumed that the bias in LOCF leads to a ‘conservative’ analysis – that is, an underestimation of treatment effects. Consider, for example, an efficacy measure. If patients drop out early – due to, say, adverse events – mean change to end-point using LOCF is assumed to lead to smaller (conservative) mean changes because patient dropout occurred before much meaningful improvement could occur. Similarly, the bias in OC is often assumed to lead to overestimation of treatment effects. Again, consider an efficacy measure. Patients who are not responding well are more likely to drop out, leaving only those patients who were responding well to complete the study.

Although these assumptions may at first look appealing, closer inspection reveals several key issues. Inferences are based upon comparisons between treatment groups, not on the change within any one group. Therefore, whether or not underestimating (overestimating) within-group changes is conservative or anticonservative depends on the relative magnitude of the bias in each treatment and on whether within-group changes represented improvement or worsening. For example, underestimating a treatment's effects might be conservative for an efficacy outcome in that we do not want to ascribe benefit to a treatment that does not in fact exist. However, underestimating a treatment's effects on a safety outcome would be anticonservative because we do not want to miss a signal regarding a potential safety risk.

Therefore, it is not surprising that analytic proofs (19,27) and studies in simulated data (12,13,21,22,26,27,29,30) have clearly shown that missing data can bias results, leading to both overestimation and underestimation of treatment effects, with the direction and magnitude of bias being difficult to anticipate and dependent on many factors. These conclusions are further substantiated by summaries of actual clinical trial data (28).

A method increasingly referred to in the literature as mixed model for repeated measures (MMRM) has key theoretical advantages over LOCF and OC (12,13,21,22,24,30,32,33). In an MMRM analysis, data collected from all patients (those who drop out as well as those who complete the study) are used to predict mean longitudinal outcomes for the treatment group. The theoretical origins of MMRM date back many decades, but this method did not receive extensive attention in the clinical trial literature until roughly the past decade, when advances in computing capabilities made MMRM easy to implement. The MMRM approach is one specific member of the larger family of likelihood-based mixed-effects analyses. This family of analyses offers a general framework from which to develop longitudinal analyses under less restrictive assumptions than LOCF and OC. The specific details of an MMRM analysis are chosen with the data characteristics of clinical trials in mind. Other likelihood-based analyses with properties similar to MMRM have been referred to in the literature as hierarchical models and random regression models. Multiple imputation (MI) is another of the more modern analytic methods, but it uses a different approach to handling missing data than MMRM. However, the theoretical underpinnings are similar, and the two methods yield similar results in actual practice (29).

The key difference between assumptions about missing data in likelihood-based analyses such as MMRM and in MI vs. the assumptions made by LOCF and OC is that MMRM and MI allow for the possibility that the observed outcomes for the variable being analysed are related to the probability of dropout. The specific assumption is referred to in the statistical literature as missing at random (MAR).

The MAR assumption is often reasonable in clinical trials as the observed data explain much of the missingness in many scenarios (4,6,12,13,19,24). This may be particularly true in well-controlled studies, such as clinical trials, in which extensive efforts are made to observe all the outcomes and the factors that influence them (16).

Regardless, MAR is always more plausible than MCAR because MAR is valid in every case when MCAR is valid, but MCAR is not always valid when MAR is valid.

Therefore, it is not surprising that numerous studies have reported that simple methods such as LOCF and OC were not as robust to the biases from missing data as MMRM and similar methods (2,5,6,10,12,13,15,19,21,22,25,26,29–31). In accordance with these findings, reviews and consensus papers from researchers with academic affiliations (31,34), consensus papers from researchers with industry affiliations (32), consensus papers from a mix of academic and industry researchers (33,35), and statistics text books (6,36) have all recommended that analyses of longitudinal clinical trial data move away from simple methods such as LOCF and OC toward the MAR-based analyses, such as MI and the likelihood-based family in which MMRM resides.

Given this fundamental shift in analytic emphasis, it is useful to characterise results from the newer, more principled methods along with those of the LOCF and OC approaches. Acute-phase efficacy results from MMRM and LOCF in actual clinical trial data have been extensively summarized (12,13,19,21,22,24–26,28,36). Hence, the purpose of this investigation was to broaden the scope of comparisons to include results from LOCF, OC and MMRM for safety and efficacy outcomes in a long-term clinical trial of patients with major depressive disorder.

## Methods

The study protocol was reviewed and approved by the ethical review board at each centre, in accordance with the principles of the Declaration of Helsinki, and all patients provided written informed consent prior to the administration of any study procedures or treatment. Results from the *a priori*-defined analyses and additional details about the design of the study used in our investigation have been reported elsewhere (37,38). Key details about the design are summarized here.

This study incorporated a double-blind, variable expected duration placebo lead-in period; followed by randomisation in a 2 : 2 : 1 ratio to fixed doses of a serotonin-noradrenalin reuptake inhibitor (SNRI), selective serotonin reuptake inhibitor (SSRI), or placebo for an 8-week, acute-treatment period; followed by a 6-month, double-blind, flexible-dose extension phase. Dose escalations for the active arms and placebo rescue via randomisation to the active treatments occurred based on predefined blinded criteria after the 8-week acute phase. The study visit schedule included efficacy and safety assessments weekly to week 4, bi-weekly to week 8 and monthly thereafter.

The trial had 684 patients with at least one post-baseline observation (SNRI, *n*= 273; SSRI, *n*= 274; placebo, *n*= 137). Study participants were outpatients, 18 years of age or older, who met Diagnostic and Statistical Manual of Mental Disorders, Fourth Edition (DSM-IV) (39) criteria for major depressive disorder (MDD), and had a Montgomery-Asberg Depression Rating Scale (40) total score ≥ 22 and a Clinical Global Impression of Severity (41) score ≥ 4 at the screening and second study visits. Exclusion criteria included a current and primary Axis I disorder other than MDD; an Axis II disorder that could interfere with protocol compliance; lack of response of the current depressive episode to two or more adequate courses of antidepressant therapy; serious medical illness; a serious risk of suicide; a history of substance dependence within the last 6 months, or a positive urine drug screen. Concomitant medications with primarily central nervous system activity were not permitted.

For this investigation, visitwise mean changes for the Maier subscale of the 17-item Hamilton Depression Rating Scale (HAMD_17_) (42) (primary efficacy outcome), blood pressure and body weight were compared using LOCF, MMRM and OC. In the LOCF analyses, missing data were imputed by carrying the last observation forward, and mean changes at each visit were assessed independently using an ancova model that included the categorical effects of treatment and investigator, with baseline value included as a covariate. In the OC analyses, the same ancova model was applied independently to the observed data at each visit. The MMRM analysis assessed data from all visits simultaneously using a restricted maximum-likelihood-based approach. The model included the fixed categorical effects of treatment, investigator, visit and treatment-by-visit interaction, with baseline value and the baseline-by-visit interaction included as covariates. Within-patient errors were modelled using an unstructured (co)variance matrix. In all analyses, placebo-treated patients were included until the visit at which they were rescued to active drug. Data from patients rescued from placebo to active drug are not included in the analyses presented.

Similar to any mean change analyses, all analyses in the present investigation assumed (approximate) normality of the residuals. The OC and LOCF analyses assumed that missing data arose from a completely random mechanism (MCAR), whereas MMRM assumed MAR. Additionally, LOCF assumed that the values for patients who discontinued would not have changed from the last observation to the end of the trial, had they stayed in the trial. In this paper ‘significant’ or ‘statistically significant’ refers to comparisons with p ≤ 0.05. All analyses were conducted using sas version 8 (SAS Institute Inc., Cary, NC, USA).

## Results

Baseline demographic and illness characteristics are summarized in Table 1. Randomisation resulted in treatment groups that did not markedly differ according to any of the assessed demographic or illness characteristics.

**Table 1:** Baseline demographics and illness severity

	**SNRI (*n* = 273)**	**SSRI (*n* = 274)**	**Placebo (*n* = 137)**
**Age, year, mean (SD)**	41.1 (11.6)*	43.3 (13.0)	42.5 (12.3)
**Age, year, range (minimum–maximum)**	18–66	18–79	20–73
**Gender, female, *n* (%)**	173 (63.4)	186 (67.9)	87 (63.5)
**Weight, kg, mean (SD)**	83.0 (20.8)	83.4 (21.8)	87.5 (24.0)
**Ethnic origin, *n* (%)**			
Caucasian	206 (75.5)	212 (77.4)	113 (82.5)
Hispanic	22 (8.1)	26 (9.5)	8 (5.8)
African-American	35 (12.8)	28 (10.2)	14 (10.2)
Asian	2 (0.7)	3 (1.1)	0 (0.0)
East Asian	3 (1.1)	1 (0.4)	0 (0.0)
Other	5 (1.8)	4 (1.5)	2 (1.5)
**HAMD**_**17**_**total score, mean (SD)**	17.6 (4.8)	17.8 (5.1)	17.7 (5.2)
**CGI-S score, mean (SD)**	4.2 (0.7)	4.2 (0.7)	4.2 (0.7)
**HAMA score, mean (SD)**	14.1 (5.2)	14.6 (5.2)	14.4 (5.1)

SNRI, serotonin-noradrenalin reuptake inhibitor; SSRI, selective serotonin reuptake inhibitor; SD, standard deviation; HAMD_17_, 17-item Hamilton Depression Rating Scale; CGI-S, Clinical Global Impression of Severity; HAMA, Hamilton Anxiety Rating Scale.

* The mean age of patients in the SNRI treatment group was statistically significantly lower than that in the SSRI group (41.1 years vs. 43.3 years; p = 0.036). There were no other significant between-group differences in baseline demographics or psychiatric profile.

**Figure 1:**
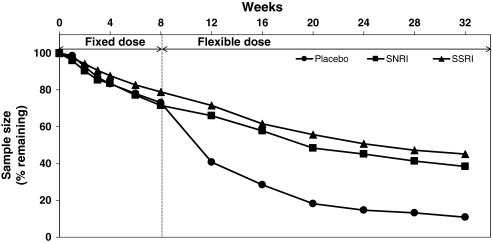
Percentage of patients remaining at each time point during the 8-month study. Flexible dosing and rescue from placebo were available after week 8. Rescue from placebo to active drug was based on investigator decision and lack of response to placebo. Data from patients rescued from placebo to active drug were analysed separately and are not presented here. SNRI, serotonin-noradrenalin reuptake inhibitor; SSRI, selective serotonin reuptake inhibitor

The change in sample size over time is shown in Figure 1. The percentage of patients completing the 8-week, acute-treatment period was 71.4% (195/273) for the SNRI, 78.8% (216/274) for the SSRI and 73.0% (100/137) for placebo. These dropout rates of 21.2 (SSRI) to 28.6% (SNRI) are not unusual for placebo-controlled, acute-treatment clinical trials in MDD, and in fact are perhaps a bit lower than the reported average dropout of 35% from the US FDA summary basis of approval reports (43). Completion percentages for the entire 8-month study were 38.5% (105/273) for the SNRI and 45.3% (124/274) for the SSRI. The impact of rescue for lack of efficacy in the placebo group was evident, as only 10.9% (15/137) completed the trial.

**Figure 2:**
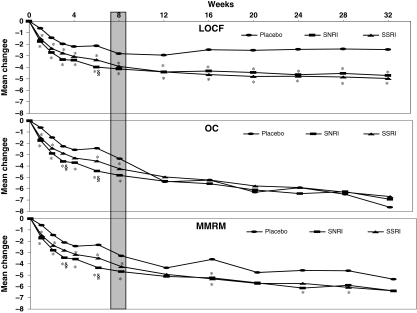
Time-course of improvement on the HAMD_17_ Maier subscale by all the three analytical methods. Double-blind placebo rescue was available after week 8. *p ≤ 0.05 vs. placebo; §p ≤ 0.05 SSRI vs. SNRI. LOCF, last observation carried forward; OC, observed case; MMRM, mixed model for repeated measures; HAMD_17_, 17-item Hamilton Depression Rating Scale

Results from the LOCF, OC and MMRM analyses for all outcomes are summarized in Tables 2 and 3, with mean changes over time for the HAMD_17_ Maier subscale (efficacy measure) depicted in Figure 2. During the acute-treatment period (through week 8), the two active-treatment arms can each be compared with placebo at six visits, yielding 12 total comparisons vs. placebo. Focusing on the mean changes (baseline to end-point) within each treatment group, LOCF tended to yield the smallest mean changes, with the greatest mean changes for OC, and MMRM being intermediate (Table 2). However, inferences are based on between-group changes, and across the 12 acute-phase comparisons, LOCF yielded 12 significant differences from placebo, compared with 10 significant differences for OC and 11 for MMRM (Figure 2). When including acute and extension periods, there are 24 opportunities to compare an active drug with placebo. With LOCF, all 24 contrasts were significant, compared with 10 for OC and 15 for MMRM. Although as suggested by Figure 2, the differences in mean changes between the drugs and placebo were slightly greater with MMRM than they were with LOCF, this was more than offset by unduly small SE from LOCF that resulted from its failure to account for the uncertainty of imputation.

**Table 2:** Summary of acute and long-term efficacy and safety outcomes by each analytical method

Assessment	LOCF, mean change (SE)	MMRM, mean change (SE)	OC, mean change (SE)
**HAMD_17_ Maier subscale, 8 weeks**			
SNRI	− 4.14 (0.23)	− 4.69 (0.24)	− 4.83 (0.25)
SSRI	− 3.92 (0.23)	− 4.24 (0.23)	− 4.24 (0.24)
**Diastolic blood pressure (mm Hg), 8 weeks**			
SNRI	+ 0.73 (0.47)	+ 1.08 (0.53)	+ 1.24 (0.53)
SSRI	− 0.85 (0.47)	− 0.80 (0.51)	− 0.79 (0.50)
**Systolic blood pressure (mm Hg), 8 weeks**			
SNRI	+ 1.26 (0.72)	+ 1.79 (0.79)	+ 2.10 (0.84)
SSRI	− 0.80 (0.71)	− 0.84 (0.76)	− 0.69 (0.80)
**Weight (kg), 8 weeks**			
SNRI	− 1.01 (0.24)	− 1.04 (0.28)	− 1.15 (0.31)
SSRI	− 0.32 (0.24)	− 0.40 (0.27)	− 0.41 (0.29)
**HAMD_17_ Maier subscale, 8 months**			
SNRI	− 4.71 (0.25)	− 6.39 (0.27)	− 6.94 (0.30)
SSRI	− 4.97 (0.25)	− 6.39 (0.25)	− 6.69 (0.27)
**Diastolic blood pressure (mmHg), 8 months**			
SNRI	+ 0.72 (0.52)	+ 0.81 (0.70)	+ 1.04 (0.84)
SSRI	+ 0.04 (0.52)	− 0.24 (0.65)	− 0.54 (0.76)
**Systolic blood pressure (mmHg), 8 months**			
SNRI	+ 2.48 (0.77)	+ 3.73 (1.14)	+ 4.10 (1.34)
SSRI	+ 0.17 (0.76)	+ 0.31 (1.06)	+ 0.01 (1.20)
**Weight (kg), 8 months**			
SNRI	0.00 (0.26)	+ 0.61 (0.44)	+ 0.76 (0.53)
SSRI	+ 1.03 (0.25)	+ 1.83 (0.42)	+ 1.22 (0.48)

LOCF, last observation carried forward; SE, standard errors; MMRM, mixed model for repeated measures; OC, observed case; HAMD_17_, 17-item Hamilton Depression Rating Scale; SNRI, serotonin-noradrenalin reuptake inhibitor; SSRI, selective serotonin reuptake inhibitor.

**Figure 3:**
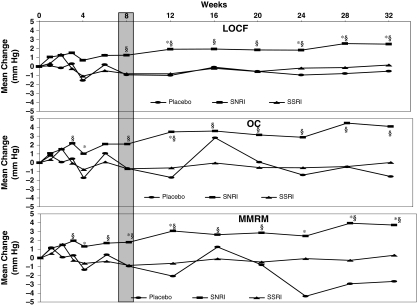
Time-course of change in systolic blood pressure by all the three analytical methods. Double-blind placebo rescue was available after week 8. *p ≤ 0.05 vs. placebo; §p ≤ 0.05 SSRI vs. SNRI

**Figure 4:**
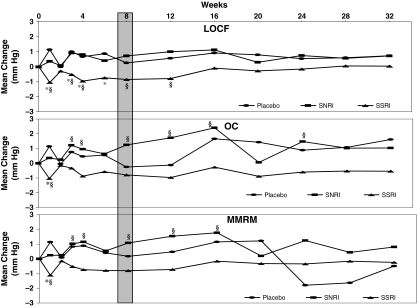
Time-course of change in diastolic blood pressure by all the three analytical methods. Double-blind placebo rescue was available after week 8. *p ≤ 0.05 vs. placebo; §p ≤ 0.05 SSRI vs. SNRI

**Figure 5:**
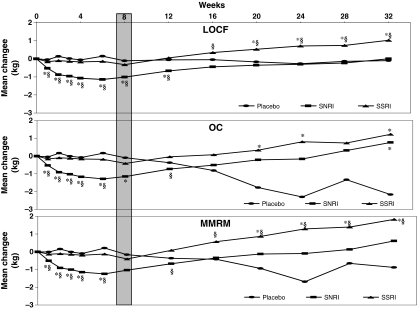
Time-course of change in body weight by all the three analytical methods. Double-blind placebo rescue was available after Week 8. *p ≤ 0.05 vs. placebo; §p ≤ 0.05 SSRI vs. SNRI

Time-courses for mean changes from LOCF, OC and MMRM analyses of systolic blood pressure, diastolic blood pressure and body weight are shown in Figures 3–5, respectively. Mean changes at week 8 and month 8 are further summarized in Table 2. Across these various safety outcomes, LOCF and MMRM generally agreed as to whether or not differences were statistically significant, with OC yielding fewer significant differences than the other methods. However, while LOCF consistently yielded the smallest within-group mean changes, the greatest within-group changes came from OC, with mean changes from MMRM being intermediate in magnitude.

These general trends are exemplified by the mean changes to month 8 for body weight that are depicted in Figure 5. Both LOCF and MMRM indicated that the mean weight increase was greater for the SSRI than for the SNRI. However, LOCF indicated no weight gain from baseline and essentially no difference from placebo at month 8 for the SNRI, and a mean increase from baseline of about 1 kg for the SSRI with a difference from placebo of 1.1 kg. In contrast, MMRM indicated a mean increase from baseline of 0.6 kg for the SNRI with a difference from placebo of about 1.5 kg, and a mean increase from baseline of 1.8 kg for the SSRI with a difference from placebo of about 2.7 kg – a difference roughly 2.5 times greater than seen with LOCF. For systolic and diastolic blood pressure, the mean increases were greater for the SNRI than for the SSRI, with the same general trend of LOCF showing smaller differences than MMRM. Observed case again tended to show the greatest within-group changes.

## Discussion

Dropout in clinical trials can arise from many factors related to the pharmacology of the drug, such as lack of efficacy, loss of initially gained efficacy due to developed tolerance, lack of initial tolerability or increased safety or tolerability issues over time. Dropout can also arise from factors not related to the drug, such as patients relocating and not being able to return to research sites. Finally, dropout can arise from unknown reasons; that is, loss to follow-up.

Therefore, the direction and magnitude of bias caused by missing data is difficult to anticipate and assess. We can only know the bias if we know the true value; and if we know the true value, we have no need to do the study. Nevertheless, missing data has been an active area of investigation for many years, and some general trends that can aid our conceptual understanding have emerged. For example, in an LOCF analysis, we assume that for patients who have dropped out, no change would have been observed from the point of dropout until end-point had those patients continued in the trial. If patients’ data would have continued to improve (or worsen) after dropout, then LOCF would underestimate the average improvement (or worsening) within a particular treatment arm.

However, inferences regarding treatment effects are based on comparisons with a control group, not on changes within a single group. Therefore, the direction and magnitude of the bias in an LOCF estimate of a treatment's effect depend on the relative bias within the treatment group compared with the control group, which in turn depends on, among other things, the rate and timing of dropout in the treatment group compared with the control group.

For example, holding all else equal, in scenarios in which the overall tendency is for improvement, such as in the acute symptomatic treatment of pain, depression, and so forth, LOCF is likely to (i) overestimate an investigational drug's advantage when dropout is higher or earlier in the comparator and underestimate its advantage when dropout is lower or later in the comparator; and (ii) overestimate the investigational drug's advantage when the advantage is maximum at intermediate time points and underestimate its advantage when the advantage increases over time. For scenarios in which the overall tendency is for worsening, such as in treating the cognitive decline of Alzheimer's disease, the above biases are reversed (44).

It is important to note the caveat in the above paragraph – holding all else constant. In actuality, the bias due to LOCF depends on many factors. For example, LOCF also assumes that the values of patients who drop out carry the same weight as the values of patients who stay in the trial. In addition, the impact of rates and timing of dropout can be enhanced or masked by rapid vs. slowly evolving changes. Therefore, the magnitude and even the direction of bias from LOCF in any one situation is extremely difficult, if not impossible, to determine.

The key assumption in an OC analysis is one of the same assumptions of LOCF: namely, that the values of patients who drop out are not different from those who stay in the trial. In many situations, patients with favourable responses are more likely to remain in the trial compared with those who have an unfavourable response. Therefore, within any one group, OC is likely to overestimate the change in favourable outcomes and underestimate the change in unfavourable outcomes. But, similar to LOCF, the bias in the estimate of the difference between treatment and control – which is the parameter of interest – depends on many factors and is difficult to assess.

In contrast to LOCF and OC, MMRM does not make assumptions about the patients that drop out. Rather, MMRM uses the actual data from all patients to predict what would have happened had patients stayed in the trial under the assumption that the data observed until the time of dropout is a useful predictor of the data that was not observed.

Some researchers have argued for the use of what is essentially an effectiveness analysis, where treatment is considered successful if some reasonable degree of improvement in efficacy is observed and if the patient completes the trial (45). The percentage of successful outcomes can be compared between treatments, and since dropout is part of the outcome, there is no argument about causal inference or imputation.

However, the primary objective of most confirmatory trials is efficacy, not effectiveness (46). Furthermore, interpretation of results from an effectiveness analysis is not clear with respect to safety outcomes. For example, assume that in the present analysis, a successful outcome on weight change is defined as gaining no more than 5% of baseline body weight and completing the study. About 90% of placebo patients would have an ‘unsuccessful’ weight change outcome because they did not complete the trial, whereas patients on the two active drugs would have higher rates of success. This result would suggest that placebo caused more weight gain than the active drugs, a result counter to clinical experience and common sense.

Results of the present investigation are generally consistent with previous reports showing that, compared with MMRM, LOCF yielded smaller within-group mean changes in efficacy (benefit) and safety (risk). OC tended to yield within-group mean changes that were greater in magnitude than MMRM. Regarding between-group changes, MMRM tended to be intermediate to LOCF and OC, with LOCF yielding the smallest between-group differences. Importantly, LOCF also tended to yield smaller SE, and therefore, especially for the efficacy outcome, yielded more statistically significant differences than the other methods. An analysis using LOCF does not distinguish between an actually observed data point and one that is imputed. Therefore, SE for LOCF mean changes at all time points are based on sample sizes as if no patients dropped out. Mathematically, this well-known bias in LOCF can result in SE that are too small because the sample size is too large, which in turn can exaggerate the statistical significance. In other words, we have more confidence in the results than we should because we think the sample size is larger than it should be. This study and others have shown more statistically significant differences in efficacy measures between treatment groups for LOCF compared with OC (47,48). Again, it is important to recognize that whether or not underestimating (overestimating) within-group changes is conservative or anticonservative depends on whether changes represent improvement or worsening.

Although it is impossible to know which method yielded results more closely reflecting the true treatment differences in this particular study, we know that MMRM is usually less biased by missing data compared with LOCF or OC. Further, the differences in results between the various methods seen in this investigation could be clinically relevant. For example, consider the mean changes in body weight at month 8. With LOCF, the differences between placebo compared with the SNRI and placebo compared with the SSRI were 0.1 and 1.1 kg, respectively. With MMRM, the corresponding differences were approximately 1.5 and 2.7 kg. That is, with LOCF, the SNRI appeared to be similar to placebo, and the SSRI had a small mean weight increase. With MMRM, the difference between placebo and the SSRI was 2.5 times the magnitude of the LOCF result. Similarly, the increase in blood pressure for the SNRI vs. placebo was twice the magnitude with MMRM compared with LOCF. MMRM, however, does not universally yield greater differences between drug and placebo compared with LOCF. At the 8-week end-point for the HAMD_17_ Maier subscale, LOCF and MMRM yielded treatment contrasts vs. placebo that were essentially identical, whereas at the 8-month end-point, contrasts vs. placebo from LOCF were greater than contrasts from MMRM.

Determining the clinical relevance of any mean change result by itself is problematic. Individual patient changes relative to baseline and, for safety end-points, absolute values relative to normal ranges must also be examined. However, the task of assessing clinical relevance is not made easier when the mean changes are potentially underestimated to the degree seen in this study for some of the LOCF safety results, or when results vary from one visit to the next as seen in some of the OC results. A more widespread use of MMRM and similar methods should improve signal detection for both efficacy and safety outcomes, thereby yielding more consistent assessments of risk and benefit.

We have attempted to illustrate some of the potential biases caused by assumptions in commonly used analytical methods using data from a real clinical trial. Perhaps the best proof of the biases in various methods, however, comes from studies involving simulated data where we know the true values. Interested readers may refer to several of the studies cited in this report for detailed examples (12,13,21,22,26,27,29,30).

Given that the present analyses were based on a single clinical study, there are some noteworthy limitations to consider. The MMRM approach has been shown across a wide variety of scenarios to be less biased by missing data than LOCF or OC; however, that does not guarantee that in every study the results from MMRM will more closely reflect the truth. Unlike in simulation studies, in this re-analysis of actual clinical trial data, we do not know the true differences between treatment groups. Therefore, it is impossible to know whether MMRM yielded results that more closely reflect the true treatment differences compared with LOCF or OC. Furthermore, this investigation is limited in that it includes only one study and may not necessarily reflect MDD trials on the whole, and may not reflect what would be seen in other disease states.

Use of MMRM is not a cure-all for the problems caused by missing data. The only sure cure for missing data is to avoid the problem altogether. Indeed, if there were no dropout, these three analytical methods (OC, LOCF and MMRM) would yield identical results. In many areas of psychiatric research, however, we accept 30–40% rates of dropout (43) in acute-phase trials as if nothing can be done. Although avoiding missing data altogether is an unattainable goal, more work is needed to understand how to design and conduct trials to reduce the rates of dropout as much as possible. In the meantime, when interpreting clinical trial results, it is important to consider rates, timing and reasons for dropout as well as the analytical methods. While we wait for further advances in analyses and trial design, it is comforting to know that use of MMRM in place of LOCF and OC is clearly a step in the right direction with regard to better estimating longitudinal treatment outcomes related to both efficacy and safety.

## Conclusion

In this study, LOCF consistently underestimated within-group changes in efficacy (benefit) and safety (risk) for both drugs compared with MMRM, and OC tended to overestimate within-group changes. However, inferences are based on between-group comparisons. Therefore, whether or not underestimating (overestimating) within-group changes was conservative or anticonservative depended on the relative magnitude of the bias in each treatment and on whether within-group changes represented improvement or worsening. Comparing results from efficacy and safety outcomes illustrated how the benefits of more robust analyses such as MMRM can improve our understanding of the risks and benefits of drugs.

**Table 3:** Summary of all statistically significant contrasts

	Week 1	Week 2	Week 3	Week 4	Week 6	Week 8	Week 12	Week 16	Week 20	Week 24	Week 28	Week 32
**HAMD_17_ Maier subscale**												
SNRI vs. placebo LOCF	*	*	*	*	*	*	*	*	*	*	*	*
SNRI vs. placebo OC	*	*	*	*	*	*						
SNRI vs. placebo MMRM	*	*	*	*	*	*		*		*		
SSRI vs. placebo LOCF	*	*	*	*	*	*	*	*	*	*	*	*
SSRI vs. placebo OC	*	*			*	*						
SSRI vs. placebo MMRM	*	*		*	*	*		*			*	
**Systolic blood pressure**												
SNRI vs. placebo LOCF							*			*	*	*
SNRI vs. placebo OC				*			*					
SNRI vs. placebo MMRM				*		*	*			*	*	*
SSRI vs. placebo LOCF												
SSRI vs. placebo OC												
SSRI vs. placebo MMRM												
**Diastolic blood pressure**												
SNRI vs. placebo LOCF												
SNRI vs. placebo OC												
SNRI vs. placebo MMRM												
SSRI vs. placebo LOCF	*		*	*	*							
SSRI vs. placebo OC	*											
SSRI vs. placebo MMRM	*											
**Weight**												
SNRI vs. placebo LOCF	*	*	*	*	*	*	*					
SNRI vs. placebo OC	*	*	*	*	*	*						*
SNRI vs. placebo MMRM	*	*	*	*	*							
SSRI vs. placebo LOCF									*	*	*	*
SSRI vs. placebo OC									*	*		*
SSRI vs. placebo MMRM									*	*	*	*
**HAMD_17_ Maier subscale**												
SSRI vs. SNRI LOCF					*							
SSRI vs. SNRI OC			*		*							
SSRI vs. SNRI MMRM			*		*							
**Systolic blood pressure**												
SSRI vs. SNRI LOCF						*	*	*	*	*	*	*
SSRI vs. SNRI OC			*			*	*	*	*	*	*	*
SSRI vs. SNRI MMRM			*		*	*	*	*	*		*	*
**Diastolic blood pressure**												
SSRI vs. SNRI LOCF	*		*	*		*	*					
SSRI vs. SNRI OC	*		*	*		*	*	*		*		
SSRI vs. SNRI MMRM	*		*	*		*	*	*				
**Weight**												
SSRI vs. SNRI LOCF	*	*	*	*	*	*	*	*	*	*	*	*
SSRI vs. SNRI OC	*	*	*	*	*		*					
SSRI vs. SNRI MMRM	*	*	*	*	*		*	*	*	*	*	*

HAMD_17_, 17-item Hamilton Depression Rating Scale; SNRI, serotonin-noradrenalin reuptake inhibitor; LOCF, last observation carried forward; OC, observed case; MMRM, mixed model for repeated measures; SSRI, selective serotonin reuptake inhibitor.

* p ≤ 0.05.
